# *Burkholderia paludis* sp. nov., an Antibiotic-Siderophore Producing Novel *Burkholderia cepacia* Complex Species, Isolated from Malaysian Tropical Peat Swamp Soil

**DOI:** 10.3389/fmicb.2016.02046

**Published:** 2016-12-21

**Authors:** Kuan Shion Ong, Yoong Kit Aw, Learn Han Lee, Catherine M. Yule, Yuen Lin Cheow, Sui Mae Lee

**Affiliations:** ^1^School of Science, Monash University MalaysiaBandar Sunway, Malaysia; ^2^Tropical Medicine and Biology Multidisciplinary Platform, Monash University MalaysiaBandar Sunway, Malaysia; ^3^Novel Bacteria and Drug Discovery Research Group, School of Pharmacy, Monash University MalaysiaBandar Sunway, Malaysia; ^4^Center of Health Outcomes Research and Therapeutic Safety (Cohorts), School of Pharmaceutical Sciences, University PhayaoPhayao, Thailand

**Keywords:** antimicrobial, *Burkholderia cepacia* complex, multilocus sequence analysis (MLSA), polyphasic taxonomy, tropical peat swamp forest

## Abstract

A novel Gram negative rod-shaped bacterium, designated strain MSh1^T^, was isolated from Southeast Pahang tropical peat swamp forest soil in Malaysia and characterized using a polyphasic taxonomy approach. The predominant cellular fatty acids (>10.0%) were C_16:0_ (31.7%), C_17:0_ cyclo (26.6%), and C_19:0_ cyclo ω8c (16.1%). The polar lipids detected were phosphatidylglycerol, phosphatidylethanolamine, and diphosphatidylglycerol. The predominant ubiquinone was Q-8. This revealed that strain MSh1^T^ belongs to the genus *Burkholderia*. The type strain MSh1^T^ can be differentiated from other *Burkholderia cepacia* complex (Bcc) species by phylogenetic analysis of 16S rRNA gene sequence, multilocus sequence analysis (MLSA), average nucleotide identity (ANI) and biochemical tests. DNA–DNA relatedness values between strain MSh1^T^ and closely related type strains were below the 70% threshold value. Based on this polyphasic study of MSh1^T^, it can be concluded that this strain represents a novel species within the Bcc, for which the name *Burkholderia paludis* sp. nov. is proposed. The type strain is MSh1^T^ (= DSM 100703^T^ = MCCC 1K01245^T^). The dichloromethane extract of MSh1^T^ exhibited antimicrobial activity against four Gram positive bacteria (*Enterococcus faecalis* ATCC 29212, *E. faecalis* ATCC 700802, *Staphylococcus aureus* ATCC 29213, *S. aureus* ATCC 700699) and a Gram negative bacteria (*Escherichia coli* ATCC 25922). Further purification work has led to the isolation of Compound 1, pyochelin. Pyochelin demonstrated antimicrobial activity against four *S. aureus* strains and three *E*. *faecalis* strains with MIC-values of 3.13 μg/ml and 6.26 μg/ml, respectively. SEM analysis showed that the cellular morphology of *E. faecalis* ATCC 700802 was not affected by pyochelin; suggesting that it might target the intracellular components. Pyochelin, a siderophore with antimicrobial activity might be useful in treating bacterial infections caused by *S. aureus* and *E. faecalis*, however further work has to be done.

## Introduction

The misuse of antimicrobial compounds to treat infections has led to an increase in the prevalence of antimicrobial resistant bacteria (ARB), often associated with nosocomial (hospital acquired) infections (Mishra et al., 2012). It was reported that nosocomial infections constitute the sixth leading cause of death in the United States and more than 70% of these nosocomial pathogens are resistant to at least one of the common antimicrobial drugs used to treat them (Iowa Department of Public Health). This is an alarming issue because ARB infections often result in increased mortality rates, limit treatment options and cause increased healthcare costs (Ammerlaan et al., 2016). Thus, there is a need for new antimicrobial compounds to combat ARB infections.

One strategy to discover new antimicrobials is bioprospecting—the exploration for new compounds in unique ecological niches (Imhoff et al., 2011). The discovery of abyssomicins, novel antibiotics produced by *Verrucosispora* sp. from South China Sea sediments is a successful example of bioprospecting (Wang et al., 2013). Bacteria producing antibiotics may have an advantage in competing for resources and colonization of new habitats in natural environments. Novel microorganisms thriving in extreme and isolated environments have the potential to develop unique biosynthesis gene clusters giving rise to novel compounds (Bull and Stach, 2007; Hibbing et al., 2010). Consequently, tropical peat swamp forests in Malaysia were chosen as potential bioprospecting locations for novel antimicrobial compounds. Tropical peat swamp forests are unique wetland ecosystems characterized by their acidic (pH range of 2.9 to 4.5) and waterlogged conditions. They are ombotrophic hence receiving nutrients solely from rain and atmospheric deposition which results in an extremely nutrient poor environment (Yule, 2010). Despite being such a harsh environment, we successfully isolated an antimicrobial-producing bacterium, belonging to the *Burkholderia cepacia* complex (Bcc) in a previous study (Ong et al., 2015).

The Bcc consists of a group of diverse, ubiquitous bacteria that occur in aquatic environments, plant rhizospheres and animals, including humans (Coenye and Vandamme, 2003; Vanlaere et al., 2009; Peeters et al., 2013). Certain Bcc species, for example *B. cepacia*, can be used for biocontrol as they can produce antifungal compounds to repress soil borne pathogens (Caballero-Mellado et al., 2004). An example of one such compound is pyrrolnitrin which targets the electron transport chain of both Gram positive bacteria and fungi (El-Banna and Winkelmann, 1998). Bcc species share a high degree of 16S rRNA (98–100%) and recA (94–95%) gene sequence similarity, and moderate levels of DNA–DNA hybridization (30–50%) (Coenye et al., 2001). In order to differentiate different species of Bcc, multi-locus sequence analysis (MLSA) are usually adopted as these taxonomic techniques provide the discriminatory power needed for both identification and differentiation of Bcc species (Spilker et al., 2009; Vanlaere et al., 2009; Peeters et al., 2013).

In this paper, we report a novel antimicrobial-producing Bcc species, isolated from the peat soil of the Southeast Pahang tropical peat swamp forest reserve in Malaysia. This polyphasic taxonomic study revealed that strain MSh1^T^ represents a novel Bcc species, for which the name *B. paludis* is proposed. Based on current literature, *Burkholderia* species are known to produce narrow-spectrum antimicrobial compounds such as iminopyrrolidines (produced by *B. plantari*) and occidiofungin (*B. contaminans*) which only targets Gram negative *Erwinia amylovora* and fungi, respectively (Lu et al., 2009; Tawfik et al., 2010). In this study, strain MSh1^T^ exhibit a different spectrum of antimicrobial activity, which targets Gram positive bacteria only, particularly strains of *S. aureus* and *E. faecalis*.

## Materials and methods

### Test microorganism strains and culture conditions

Test microorganism strains that were used in this study include *Bacillus cereus* ATCC 14579, *Bacilus subtilis* ATCC 8188, *Enterococcus faecalis* ATCC 700802, *Enterococcus faecalis* ATCC 29212, *Enterococcus faecalis* JH-22, *Staphylococcus aureus* ATCC 700699, *Staphylococcus aureus* ATCC 43300, *Staphylococcus aureus* ATCC 6538P, *Staphylococcus aureus* ATCC 29213, *Aeromonas hydrophila* ATCC 49140, *Escherichia coli* ATCC 25922, *Klebsiella pneumoniae* ATCC 10031, *Proteus mirabilis* ATCC 49140, *Proteus vulgaris* (Institute of Medical Research, Malaysia), *Pseudomonas aeruginosa* ATCC 10145, *Pseudomonas aeruginosa* ATCC BAA-47, *Salmonella* Typhimurium ATCC 14028, and *Shigella flexneri* ATCC 12022. Strains were cultured on Muller Hinton borth (MHB) (Oxoid, UK) at 37°C and maintained at −80°C in MHB with 25% (v/v) glycerol.

### Isolation and maintenance of isolate

Strain MSh1^T^ was previously isolated from surface peat collected from the Southeast Pahang tropical peat swamp forest reserve (3° 01′ 19.56″ N; 103° 39′ 29.67″ E) in Malaysia on March 2013. Strain MSh1^T^ was maintained on nutrient agar (NA) at 30°C and in 25% (v/v) glycerol in nutrient broth (NB) (Merck, Germany) at −80°C for long term preservation.

### Sequence and phylogenetic analysis

The genomic DNA of strain MSh1^T^ was isolated using GF-1 nucleic acid extraction kit (Vivantis, Malaysia). The 16S rRNA gene sequence of strain MSh1^T^ was amplified using the universal primers 27f (5′-AGA GTT TGA TCC TGG CTC AG-3′) and 1492r (5′-ACG GCT ACC TTG TTA CGA CTT 3′) (Kane et al., 1993). PCR were set up as follows: 150 ng (5 μL) of DNA extract, 10 μL of 5x MyTaq Red Reduction Buffer, 5 μM of forward primer, 5 μM of reverse primer, and 1.25 U of MyTaq DNA polymerase. The reaction volume was made up to 50 μL using sterile milliQ water (Millipore, Germany). The PCR included an initial denaturation step at 95°C for 1 min, followed by 30 cycles of denaturation at 95°C for 15 s, annealing at 60°C for 45 s and elongation at 72°C for 45 s. PCR products were separated on a 1.5% (w/v) agarose gel and the bands were visualized with 1x GelRed. Amplification products were purified and cloned into pJET1.2 vector (FirstBase, Malaysia). The 16S rRNA gene sequence of strain MSh1^T^ was aligned with sequences of closely related type strains of the genus *Burkholderia* retrieved from the GenBank/EMBL/DDBJ databases using CLUSTAL-X software (Thompson et al., 1997). The alignment was manually verified and adjusted prior to the construction of phylogenetic tree using the neighbor-joining (Saitou and Nei, 1987) algorithm with the MEGA version 6.0 software (Tamura et al., 2011). The stability of the resultant tree topologies were evaluated by using the bootstrap resampling method (Felsenstein, 1985). The evolutionary distances for the neighbor-joining algorithm were computed using the Kimura's two-parameter model (Kimura, 1980).

### DNA–DNA hybridization (DDH)

DDH-tests were carried out by the Identification Service of Deutsche Sammlung von Mikroorganisen und Zellkulturen (DSMZ, Germany) to evaluate the DNA–DNA relatedness between strain MSh1^T^, *B. arboris* R-24201^T^, *B. cenocepacia* J2315^T^, and *B. lata* 383^T^ using the optical renaturation rate method as described by De Ley et al. (1970) under consideration of the modifications described by Huss et al. (1983).

### Multilocus sequence analysis (MLSA)

MLSA was performed on strain MSh1^T^ based on the method described by Spilker et al. (2009). A phylogenetic tree of the concatenated sequence (2773 bp) of seven housekeeping gene fragments [*atp*D (443 bp), *glt*B (400 bp), *gyr*B (454 bp), *rec*A (393 bp), *lep*A (397 bp), *pha*C (385 bp) and *trp*B (301 bp)] was constructed using MEGA6.0 (Tamura et al., 2011). The mean number of nucleotide substitution per site (i.e., the percentage of divergence of concatenated allele sequences) between established Bcc type strains and strain MSh1^T^ was calculated using the DnaSP v5.10 (Librado and Rozas, 2009) based on the Jukes-Cantor method (Jukes and Cantor, 1969). Nucleotide sequences of each allele, allelic profiles and sequence types of strain MSh1^T^ from the present study are available on the Bcc PubMLST database (http://pubmlst.org/bcc).

### PCR amplification of *prnD* (pyrrolnitrin) gene

The synthesis of pyrrolnitrin is regulated mainly by four genes *prnA, prnB, prnC* and *prnD*, where *prnD* being the most crucial gene. The gene product of *prnD* catalyzes the oxidation of the amino group of aminopyrrolnitrin to a nitro group to form pyrrolnitrin (Souza and Raaijmakers, 2003). As pyrrolnitrin is the only reported compound with antimicrobial activity against Gram positive bacteria known to be produced by Bcc (El-Banna and Winkelmann, 1998), this assay was carried out to avoid the rediscovery of a known compound (pyrrolnitrin). Hence in order to assess the ability of strain MSh1^T^ to produce pyrrolnitrin, the *prnD* gene was amplified with a forward primer PRND1 (5′-GGG GCG GGC CGT GGT GAT GGA-3′) and a reverse primer PRND2 (5′-YCC CGC SGC CTG YCT GGT CTG-3′) (Souza and Raaijmakers, 2003). PCR were set up as follows: 5 μL of DNA extract, 10 μL of 5x MyTaq Red Reduction Buffer, 5 μM of forward primer, 5 μM of reverse primer, and 1.25 U of MyTaq DNA polymerase. The reaction volume was made up to 50 μL using sterile milliQ water (Millipore, Germany). The PCR included an initial denaturation step at 95°C for 1 min, followed by 30 cycles of denaturation at 95°C for 60 s, annealing at 68°C for 30 s, and elongation at 72°C for 30 s. Genomic DNA of pyrrolnitrin producer *B. lata* 383^T^ was used as a positive control and non-pyrrolnitrin producer *B. cenocepacia* J2315^T^ was used as a negative control. PCR products were separated on a 1.5% (w/v) agarose gel and the bands were visualized with 1x GelRed.

### Phenotypic and biochemical characterization

Cellular morphological characteristics of strain MSh1^T^ were observed by using a Hitachi S-4800 field-emission scanning electron microscopy (FE-SEM) at 25,000 × magnification after 2 days of growth in nutrient broth (NB) at 30°C. Colony morphology of strain MSh1^T^ was examined after 2 days of growth on NA at 30°C. Cell motility was measured using hanging drop method after incubation for 2 days at 30°C in NB (Robbie, 1945). The optimal temperature for growth was measured at 4, 15, 28, 30, 37, 40, and 45°C in NB for 2 days. The pH range for growth was tested with in NB adjusted with HCl or NaOH to pH 3–11, at intervals of 0.5 pH units. NaCl tolerance at 0–5% (w/v) at interval of 0.5% (w/v) was determined in NB for 2 days. Anaerobic growth was tested in NB for up to 5 days in a 2.5 l jar containing an AnaeroPack-Anaero (Thermo Scientific, USA). Phenotypic characteristics including Gram staining, catalase, and oxidase activity were examined using the methods described by Buck (1982) and Cappuccino and Sherman (2002) after 2 days of growth on NA at 30°C. Physiological and biochemical properties were further determined using API 50CH, API 20NE, and API ZYM strips (bioMerieux, France) according to the manufacturer's instructions. All tests were conducted in duplicate. The API 50CH and API 20NE-tests were read after 24–48 h at 30°C, while the API ZYM-tests were read after 4 h of incubation at 30°C.

### Chemotaxonomic characterization

The cellular fatty acids analysis of strain MSh1^T^, *B. arboris* R-24201^T^, *B. cenocepacia* J2315^T^, and *B. lata* 383^T^ was carried out by the Identification Service of DSMZ (Braunschweig, Germany). The cell mass of strain MSh1^T^ and closely related type strains were harvested from NB after incubation at 30°C for 2 days. Extraction and analysis of the cellular fatty acids were performed according to the standard protocols of the Sherlock Microbial Identification System (MIDI) (Miller, 1982), analyzed using an Agilent 6890N gas chromatograph fitted with a 5% phenyl-methyl silicone capillary column. Peaks were integrated and identified using the peak-naming table TSBA40 at the DSMZ. Polar lipids extracted from 200 mg freeze-dried cell material using chloroform: methanol: 0.3% (w/v) aqueous NaCl mixture 1:2:0.8 (v/v/v) as described by Tindall (1990). The extracted polar lipids were then separated by two dimensional silica gel thin layer chromatography with chloroform: methanol: water (65:25:4, v/v/v) as mobile phase for the first direction, followed by chloroform: methanol: acetic acid: water (80:12:15:4, v/v/v/v) as the mobile phase for the second direction (Tindall et al., 2007). Cellular ubiquinones were extracted and purified as described previously by Tindall (1990). The different quinone classes were firstly separated using thin layer chromatography on silica gel using hexane-tert-butylmethylether (9:1, v/v) as a solvent and then further purified with reversed-phase HPLC using methanol:heptane (9:1, v/v) as the eluent. The purified quionones were compared to standards at the DSMZ.

### Genome-to-genome distance calculator (GGDC) and average nucleotide identity (ANI)

GGDC was performed to predict the DNA–DNA hybridization value between strain MSh1^T^ with related *Burkholderia* species. GGDC was performed at http://ggdc.dsmz.de/distcalc2.php using the standard parameters (GGDC 2.0 BLAST+) (Meier-Kolthoff et al., 2013). The genome sequence of strain MSh1^T^ (DDBJ/EMBL/GenBank JPGL00000000) (Ong et al., 2014) was queried with related Bcc species. Results were expressed as similarity percentage ± confidence interval. ANI was performed to estimate the mean values between homologous genomic regions shared by strain MSh1^T^ with related Bcc species using the whole genome sequence obtained. ANI was performed at http://enve-omics.ce.gatech.edu/ani using the standard parameters (Goris et al., 2007). Results were expressed as ANI percentage ± standard deviation.

### Genome analysis of secondary metabolites gene clusters using antismash

The whole genome of strain MSh1^T^ was screened for biosynthetic gene clusters responsible for the synthesis of secondary metabolites using Antibiotics and Secondary Metabolite Analysis Shell (antiSMASH) 2.0) (Blin et al., 2013). The antiSMASH 2.0 program analyses the whole genome sequence for homologs to known secondary metabolites via BLAST search and annotates them based on different biosynthetic gene clusters, for example polyketide synthase (PKS), non-ribosomal peptide synthase (NRPS), terpene and bacteriocins.

### Methanol extraction of MSh1^T^

Strain MSh1^T^ was first grown on nutrient agar supplemented with 5 g/l of glycerol for 5 days at 30°C. The whole agar including the bacteria was extracted three times with 100% methanol (Merck, Germany) (Isnansetyo and Kamei, 2003). The crude methanol extract was lyophilized using a Freezone 4.5 Plusfreeze Dryer (Labconco, USA).

### Sequential solvent fractionation and purification of the crude methanol extract

Sequential solvent fractionation was performed on the crude methanol extract to fractionate the extract into different fractions with different polarity. Five grams of crude methanol extract was first dissolved in 500 ml of distilled water, and then extracted with hexane (HEX), dichloromethane (DCM) and ethyl acetate (EtOAc) sequentially. Each extraction step was performed three times and combined into one fraction before lyophylization in a Freezone 4.5 Plusfreeze dryer (Labconco, USA). The lyophilized active fraction was dissolved in methanol and partially purified on an open C_18_ column (Merck, Germany), followed by reversed-phase HPLC using a Cosmosil 5C18-MS-II, 20 × 250 mm, 5 μm column (Nacalai, USA), to yield compound 1. All processes were monitored by bioassay.

### Identification of the antimicrobial compound(s) from strain MSh1^T^

Structural determination of the antimicrobial compound was performed by spectroscopic techniques and literature comparison. The antimicrobial compound was analyzed by thin-layer chromatography on a silica 60 plates (Merck, Germany) with chloroform-acetic acid-ethanol at 95:5:2.5 (v/v) as the mobile phase, followed by spraying of an iron reagent (0.1 M FeCl_3_ in 0.1 M HCl). LC-MS was performed with an Agilent 1290 Infinity LC system coupled to Agilent 6520 Accurate-Mass Q-TOF mass spectrometer (dual ESI source) equipped with an Agilent Zorbax Eclipse XDB-C18 column. The ultraviolet/visible absorption spectrum was recorded with the photodiode array detector equipped with the above-mentioned HPLC. The mobile phase was composed of water (A, 0.5% formic acid) and acetonitrile (B, 0.5% formic acid), the gradient program of which was 0–12.00 min 90% A and 10% B and 12.00–15.00 min 100% B. The follow rate of the mobile phase was 0.3 ml/min, and the column temperature was set to 25°C. The injection volume was 10 μl.

### Antimicrobial testing via broth microdilution

The antimicrobial activity of each fraction was evaluated using a broth microdilution assay to determine the minimum inhibitory concentration (MIC) of an antimicrobial compound as described by the Clinical and Laboratory Standard Institute (CLSI). MIC is defined as the lowest concentration of an antimicrobial to inhibit the visible growth of a microorganism after overnight incubation. Briefly, the test microorganisms were grown in MHB at 37°C for 24 h and adjusted to 0.5 McFarland standard (OD_625_ 0.08–0.11). The adjusted cultures were then diluted 100 times in MHB and used as inocula. The extracts were serially diluted using sterile MHB in a 96-well flat bottomed microtiter plate. One hundred micro liters of test microorganisms corresponding to approximately 10^5^ colony forming units (CFU) was added to each well. Determination of MIC was performed in triplicate. The positive control for bacteria was 100 μg/ml chloramphenicol. The negative control contained MHB with test microorganisms. The blank control consisted only of MHB. The microtiter plate was incubated at 37°C aerobically for 24 h and the MIC was determined by the concentration of extract (μg/ml) where no visible growth was observed.

### Scanning electron microscopy (SEM)

SEM was performed based on the method described by Pilsczek et al. (2010) with modification, to determine the effect of the extract on the cellular morphology of the bacteria. *E. faecalis* ATCC 700802 was grown in MHB at 37°C for 24 h and the turbidity was adjusted to 0.5 McFarland standard. The MIC of the extract was added to the adjusted bacterial culture. An untreated control was used as a negative control, while treatment with 100 μg/mL chloramphenicol was used as the positive control. All samples were incubated at 37°C for 4 h. The cultures were then centrifuged at 5000 × *g* for 3 min and the supernatant was discarded. The bacterial pellet was washed with phosphate buffered saline (PBS) and subjected to centrifugation at 5000 × g for 3 min. This washing process was repeated three times. The washed pellets were reconstituted in minimal volume of PBS, placed onto glass slides (5 × 5 mm) and allowed to air dry for 30 min. The slides were fixed using 2.5% (v/v) gluteraldehyde in PBS for 4 h and washed three times with PBS to remove excess glutaraldehyde. The slides were then serially dehydrated with increasing concentration of ethanol and kept in a desiccator overnight. The slide was spur-coated with platinum using Q150R rotary-pumped sputter coater before observed using SU8010 FE-SEM (Hitachi, Japan).

### Nucleotide sequence accession number

The 16s rRNA gene sequence of strain MSh1^T^ has been deposited in GenBank/EMBL/DDBJ under the accession number KT159931. The gene sequences of each MLSA loci have been deposited at the Bcc PubMLST database with sequence typing (ST) 1043; and GenBank/EMBL/DDBJ under the accession number KU301866–301872.

## Results

### 16S rRNA gene sequence analysis

**Figure 1:**
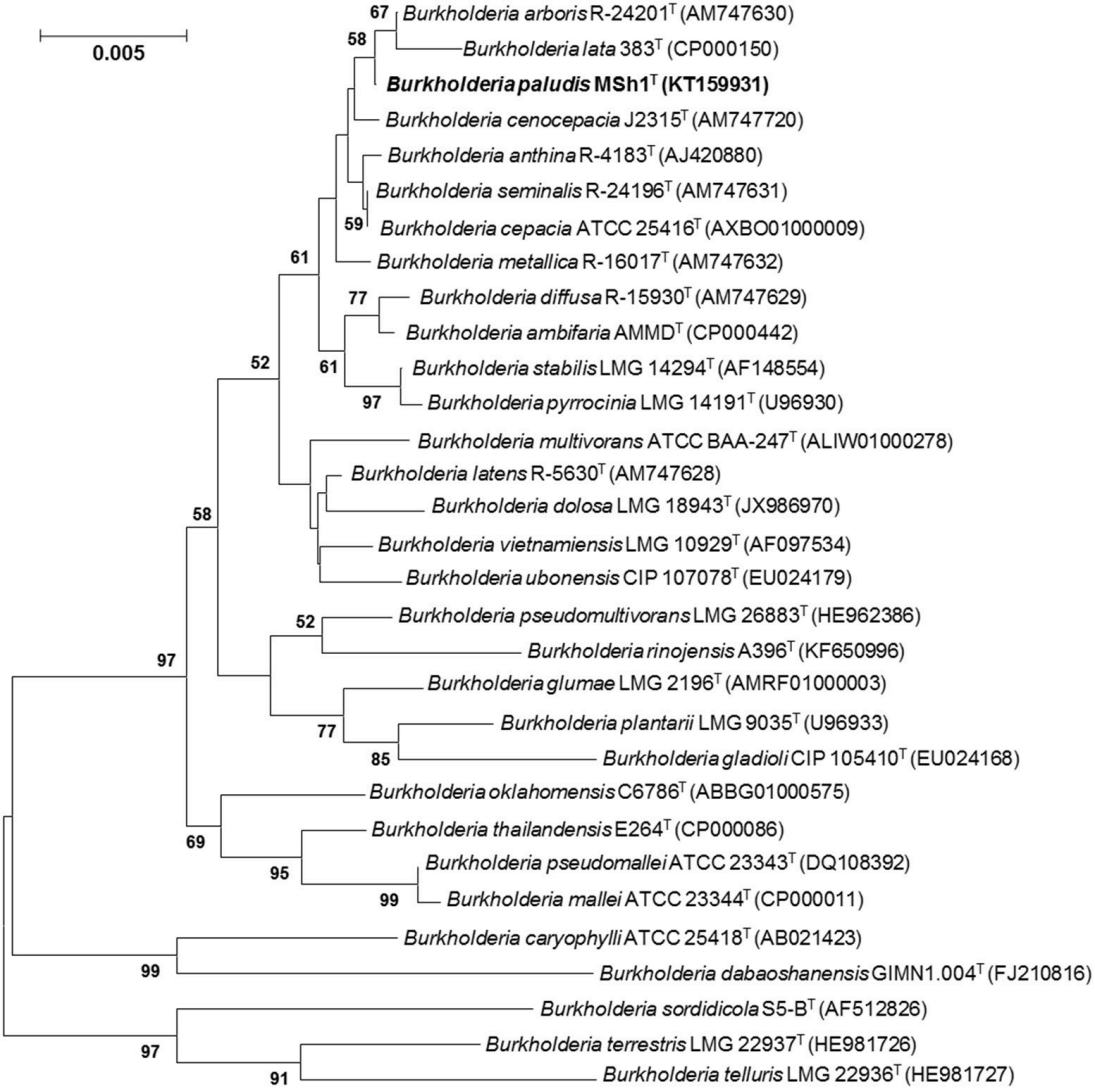
**Neighbor-joining tree based on 16S rRNA sequences showing relationship between strain MSh1^T^ and representatives of some other related taxa**. Bootstrap values (>50%) based on 1000 resampled datasets are shown at branch nodes. Bar, 5 substitutions per 1000 nucleotide positions.

The 16S rRNA gene sequence was obtained for strain MSh1^T^ (1497 bp; GenBank/EMBL/DDBJ accession number KT159931) and a phylogenetic tree was constructed (Figure 1). Phylogenetic analysis demonstrated that strain MSh1^T^ is closely related to *B. arboris* R-24201^T^ and *B. lata* 383^T^, as they formed a distinct clade (Figure 1). Pairwise comparison of the 16S rRNA gene sequence of strain MSh1^T^ with those Bcc type strains revealed similarity levels between 97.1 and 99.9% (data not shown).

### DDH

The DNA–DNA relatedness values between strain MSh1^T^ with its close neighbor based on 16S rRNA phylogenetic analysis: *B*. *arboris* R-24201^T^ (29.0 ± 3.7%), *B. cenocepacia* J2315^T^ (32.8 ± 2.2%) and *B. lata* 383^T^ (19.5 ± 1.8%) were significantly below the 70% threshold value for species delineation (Wayne et al., 1987).

### MLSA

**Figure 2:**
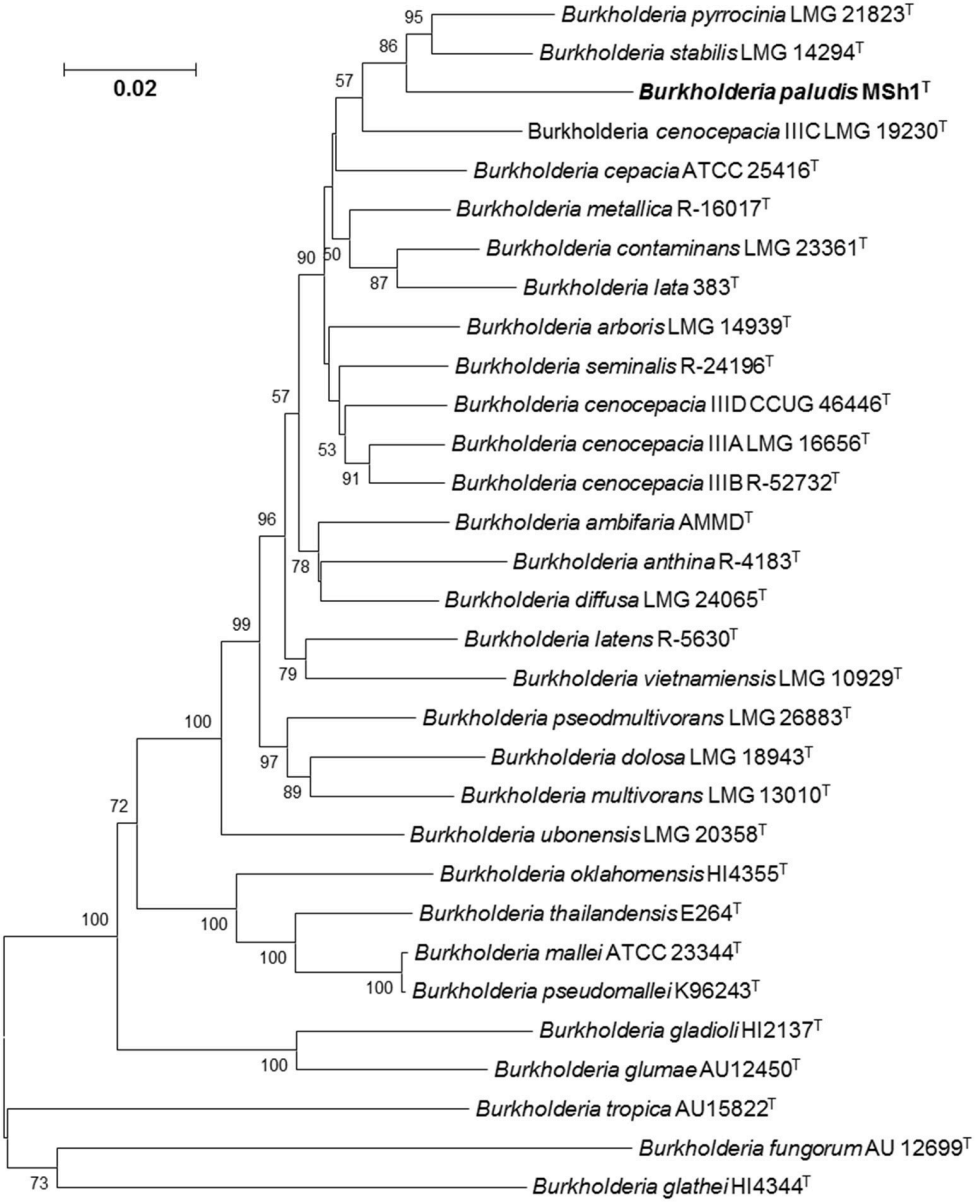
**Phylogenetic tree based on the concatenated sequences of seven housekeeping gene fragments of established Bcc species and strain MSh1^T^**. Bootstrap values (>50%) based on 1000 resampled datasets are shown at branch nodes. Bar, 2 substitutions per 100 nucleotide positions.

Phylogenetic analysis of concatenated allele sequences demonstrated that strain MSh1^T^ diverged from the other closely related Bcc type strains, supported by a bootstrap value of 86% (Figure 2). MLSA data were used to assign allele types and allelic profiles. The allelic profile were as follows: *atpD*, 302; *gltB*, 11; *gyrB*, 50; *recA*, 350; *lepA*, 288; *phaC*, 249; and *trpB*, 345 (GenBank/EMBL/DDBJ accession number KU301866–301872). Strain MSh1^T^ showed distinct allelic profile when compared with those closely related type strains (Table S1). For each established Bcc species, all allele types and allelic profiles were exported from the Bcc MLST database. The average concatenated allele sequence divergence of strain MSh1^T^ toward its nearest neighbor *B. stabilis* LMG 14294^T^ (5.23%), *B. pyrrocinia* LMG 14191^T^ (5.81%) and *B. cenocepacia* IIIC 19230^T^ (6.87%) were above the 3% cut-off value hence indicating that strain MSh1^T^ is a novel species within the Bcc (Table S2) (Vanlaere et al., 2009; Peeters et al., 2013). Moreover, these data further substantiated the results obtained from DDH which confirmed that strain MSh1^T^ is a novel species within the Bcc as the concatenated allele divergences between strain MSh1^T^ and its close neighbor, *B. arboris* R-24201^T^, *B. cenocepacia* J2315^T^ and *B. lata* 383^T^ were more than 3%.

### Phenotypic and biochemical characterization

**Figure 3:**
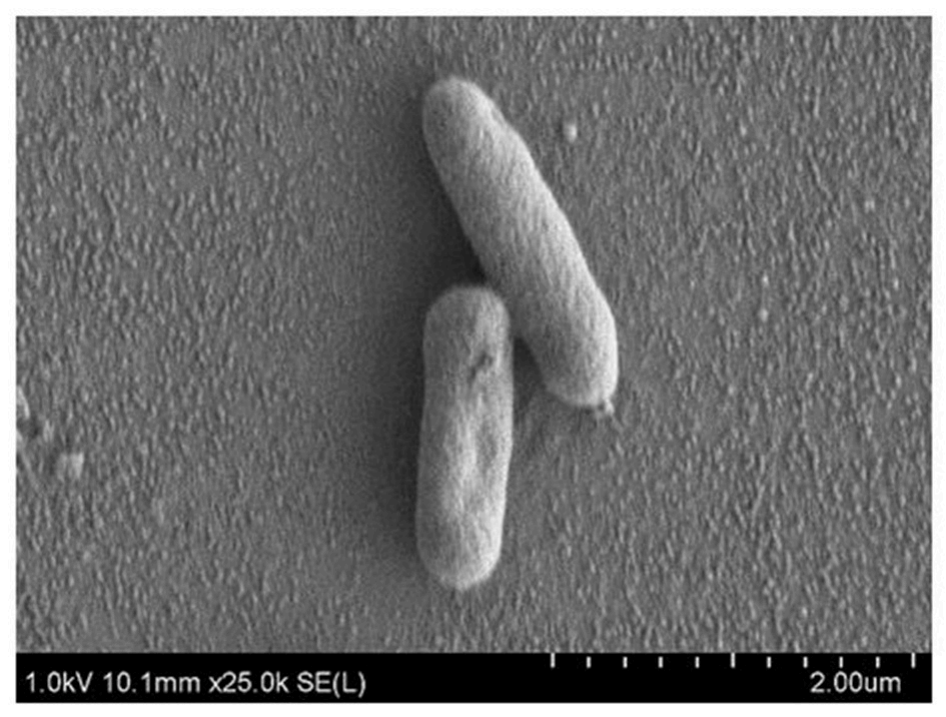
**SEM images of *Burkholderia paludis* MSh1^T^**. Images were taken under 25,000 × magnification at 1 kV. The bacterial cells sizes are approximately 0.6–0.8 × 1.6–2.1 μm.

Strain MSh1^T^ was Gram negative, facultative anaerobic, motile, rod-shaped, 0.6–0.8 × 1.6–2.1 μm bacterium (Figure 3). Colonies produced by the strain MSh1^T^ on NA were round, yellow colored with a smooth surface and 2–3 mm in diameter. Growth of strain MSh1^T^ occurs at 15–40°C (optimum 30°C) and pH 4.0–10.0 (optimum pH 7.0) in NB. Growth occurs with 0–2.5% NaCl (optimum without NaCl). Table 1 shows that strain MSh1^T^ can be differentiated biochemically from the closely related members of the genus *Burkholderia*. It was shown that strain MSh1^T^ differs from the other Bcc species by the ability to acidify adonitol, having arginine dihydrolase activity and inability to assimilate N-acetyl-glucosamine (Vandamme et al., 1997, 2000, 2002; Coenye et al., 2001; Henry et al., 2001; Vanlaere et al., 2008, 2009; Peeters et al., 2013).

**Table 1:** **Differentiation characteristics of strain MSh1^T^ and type strains of closely related species of Bcc**.

**Characteristics**	**1**	**2**	**3**	**4**	**5**	**6**	**7**	**8**	**9**	**10**	**11**	**12**	**13**	**14**	**15**	**16**	**17**	**18**	**19**	**20**
**ACIDIFICATION OF**																				
Sucrose	+	+	+	–	+	–	+	–	+	v	+	+	+	+	+	+	+	–	–	–
Adonitol	–	+	+	+	v	+	–	+	+	v	+	–	+	v	+	+	+	+	+	+
**ASSIMILATION OF**																				
N-acetyl-glucosamine	–	+	+	+	+	+	+	+	+	+	+	+	+	+	+	+	+	+	+	+
**ENZYMATIC ACTIVITY OF**																				
Arginine decarboxylation	+	–	–	–	–	–	–	–	–	–	–	+	–	–	–	–	–	–	–	–
Lysine decarboxylation	+	+	+	+	+	+	+	–	+	v	+	–	+	+	v	+	+	v	+	–
β-galactosidase	–	+	+	–	+	–	+	+	+	v	+	–	+	+	+	+	+	+	+	+
**FATTY ACID CONTENT**																				
C_16:0_(%)	31.7	29.8	36.5	28.2	26.8	25.6	19.5	26.1	26.3	28.4	22.9	16.6	17.8	21.2	28.1	26.4	21.9	28.9	20.9	29.0
C_17:0_cyclo (%)	26.6	22.5	26.1	23.5	17.9	17.8	14.0	16.0	11.3	4.6	13.5	5.7	12.6	1.6	11.7	10.4	5.2	18.2	8.0	17.2
C_19:0_ cyclo ω8c (%)	16.5	13.7	17.6	19.8	12.5	15.3	5.8	14.8	4.8	1.3	4.7	2.4	9.5	0.4	1.6	3.0	1.8	9.7	4.2	10.0

*Species: 1, strain MSh1^T^; 2, B. arboris R-24201^T^; 3. B. cenocepacia J2315^T^; 4, B. lata 383^T^; 5, B. cepacia; 6, B. stabilis; 7, B. vietnamiensis; 8, B. dolosa; 9, B. ambifaria; 10, B. anthina; 11, B. pyrrocinia, 12, B. ubonensis; 13, B. latens; 14, B. diffusa; 15, B. seminalis; 16, B. metallica; 17, B. contaminans; 18; B. multivorans; 19, B. pseudomultivorans; 20, B. gladioli*.

*Data for strain MSh1^T^, B. arboris R-24201^T^, B. cenocepacia J2315^T^, and B. lata 383^T^ are from this study. Data for the other Bcc species were obtained from Coenye et al. (2001), Henry et al. (2001), Peeters et al. (2013), Vandamme et al. (2002), Vandamme et al. (1997), Vandamme et al. (2000), Vanlaere et al. (2009), and Vanlaere et al. (2008). Data for fatty acid content are percentages of whole-cell fatty acid content. +, >90% of all isolate positive; v, 10–90% position; –, <10% of strains positive*.

### Chemotaxonomic characterization

**Figure 4:**
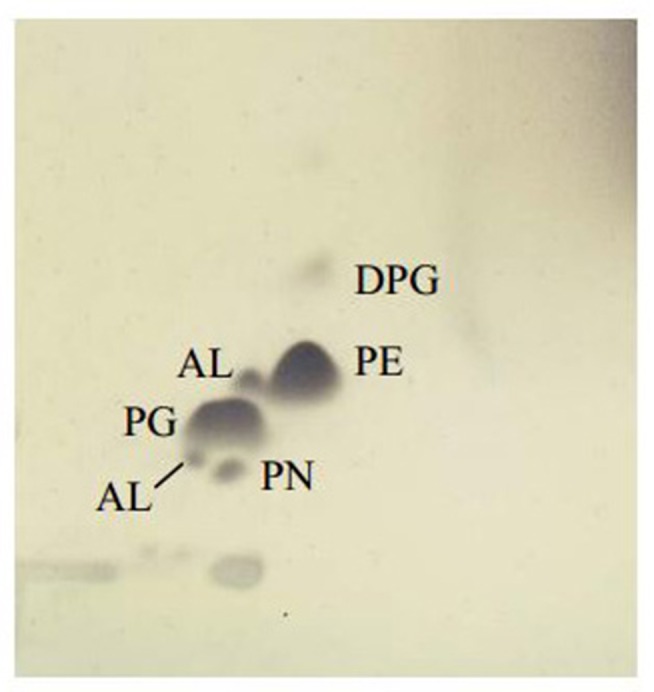
**Thin layer chromatogram polar lipid detected in *Burkholderia paludis* MSh1^T^**. AL, aminolipid; PN, aminophospholipid; PG, phosphatidylglycerol; PE, phosphatidylethanolamine; DPG, diphosphatidylglycerol.

Chemotaxonomic analysis revealed that the major cellular fatty acids were C_16:0_ (31.7%), C_17:0_ cyclo (26.6%) and C_19:0_ cyclo ω8c (16.1%). The fatty acid profile of MSh1^T^ was consistent with these of closely related type strains such as *B. arboris* R-24201^T^, *B. cenocepecia* J2315^T^, and *B. lata* 383^T^ which contained fatty acid C_16:0_ (28.2–36.5%), C_17:0_ cyclo (22.5–26.1%) and C_19:0_ cyclo ω8c (13.7–19.8%) (Table 1). The ubiquinone Q-8 was detected. The polar lipids consisted phosphatidylglycerol (PG), phosphatidylethanolamine (PE) and diphosphatidylglycerol (DPG), aminolipid and aminophospholipid (Figure 4). The fatty acid profile, polar lipid profile and major isoprenoid quinone of strain MSh1^T^ were consistent with *Burkholderia* type strains (Gillis et al., 1995).

### Genome-to-genome distance calculator (GGDC) and average nucleotide identity (ANI)

GGDC is an *in silico* genome-to-genome comparison tool used to calculate the intergenomic distances and relatedness of strain MSh1^T^ with known type strains (Meier-Kolthoff et al., 2013). The data obtained is converted to similarity values analogous to DNA–DNA hybridization (DDH) hence similarity value of 70% is generally regarded as a threshold for new species determination (Meier-Kolthoff et al., 2013). It was found that the similarity values between strain MSh1^T^ with related known Bcc species are less than 70%, thus suggesting that this isolate might represent new species. This is further supported by the ANI results which revealed that strain MSh1^T^ had ANI-value lower than the 96% threshold for new species (Goris et al., 2007; Table S3).

### Genome analysis of secondary metabolites gene clusters using antismash

Antimicrobial compounds produced by bacteria are usually secondary metabolites which are regulated by biosynthetic gene clusters. These gene clusters can be annotated using antiSMASH 2.0 (Blin et al., 2013, 2014). Analysis showed that the genome of strain MSh1^T^ had 43 gene clusters responsible for the biosynthesis of secondary metabolites which include the PKS, NRPS, bacteriocin, and terpene biosynthetic genes. Besides that, it was found that strain MSh1^T^ (43 gene clusters) possess a higher number of biosynthetic gene clusters compared to other related *Burkholderia* species (9 to 25 gene clusters) despite having similar genome size (Table 2). This might indicate that some of the putative biosynthetic gene clusters might be new as antiSMASH was unable to annotate due to the lack of similarity between query sequences from the database. These results revealed that the antimicrobial compounds produced by strain MSh1^T^ might either be new or under-studied.

**Table 2:** **Comparison of gene clusters responsible for secondary metabolites biosynthesis between strain MSh1 with related *Burkholderia* species**.

***Burkholderia* strains**	**Genome size (Mbp)**	**PKS**	**NRPS**	**Hybrid**	**Bacteriocin**	**Putative**	**Others**	**Total**
*Burkholderia* sp. MSh1	8.63	1	1	1	4	29	7	43
*B. ambifaria* AMMD	7.64	1	2	2	2	0	12	19
*B. cenocepacia* J2315	8.06	1	3	0	1	0	8	13
*B*. *cepacia* GG4	6.47	1	1	0	1	0	5	8
*B. lata* sp. 383	8.68	1	2	0	2	0	8	13
*B. multivorans* ATCC 17616	7.01	1	1	0	1	0	10	13
*B. pyrrocinia* CH-67	8.04	1	3	2	3	0	10	19
*B. ubunensis* Bu	6.93	0	4	1	3	0	17	25
*B. vietnamiensis* G4	8.39	0	1	0	1	0	7	9

*PKS, polyketide synthase; NRPS, non-ribosomal polyketide synthethase; hybrid, combination of PKS and NRPS*.

### Antimicrobial activity of MSh1^T^

The methanol extract of MSh1^T^ demonstrated antimicrobial activity four Gram positive bacteria and a Gram negative bacterium (Table 3). It was found that the Gram positive bacteria had lower MIC-value when compared to the Gram negative bacteria. Sequential solvent fractionation of the methanol crude extract yielded four different fractions with different polarities (HEX fraction, DCM fraction, EtOAc fraction and water fraction). The DCM fraction showed similar antimicrobial activity (MIC 0.020 mg/ml) to the crude methanol extract, indicating that the antimicrobial compounds had successfully been fractionated from the original extract. Further purification of the DCM extract has led to the isolation of Compound 1, pyochelin. Antimicrobial testing of Compound 1 was performed with 13 other test microorganisms via broth microdilution. Compound 1 demonstrated antimicrobial activity against four *Staphylococcus* strains and three *Enterococcus* strains; but not *Bacillus subtilis* ATCC 8188, *B. cereus* ATCC 14579 and all other Gram negative bacteria tested. SEM analysis showed that the cellular morphology of *E. faecalis* ATCC 700802 was not affected by pyochelin.

**Table 3:** **The minimum inhibitory concentration (MIC) of different fractions and crude methanol extract against the five test microorganism strains**.

**Test microorganism**	**MIC (mg/ml)**				
	**CME**	**HEX**	**DCM**	**EtOAc**	**Water**
*E. faecalis* ATCC 29212	0.020	>5.000	0.020	0.313	0.156
*E. faecalis* ATCC 700802	0.039	>5.000	0.039	0.313	0.313
*S. aureus* ATCC 29213	0.313	>5.000	0.313	2.500	1.250
*S. aureus* ATCC 700699	0.156	>5.000	0.156	1.250	1.250
*E. coli* ATCC 25922	2.500	>5.000	2.500	1.250	1.250

*CME, Crude methanol extract; HEX, Hexane fraction; DCM, Dichloromethane fraction; EtOAc, Ethyl acetate*.

## Discussion

The taxonomic position of strain MSh1^T^ was determined and characterized using a polyphasic approach. The 16S rRNA sequence of strain MSh1^T^ revealed that it belonged to the genus *Burkholderia* and was closely related to the Bcc. Phylogenetic analysis further showed that strain MSh1^T^ clades closely to *B. arboris, B. lata* and *B. cenocepacia*. Despite having high 16S rRNA gene sequence similarity (data not shown), phylogenetic analysis indicated that strain MSh1^T^ is situated on a different branch in the tree, signifying the possibility of being a novel species. As members of Bcc are known to have high similarity in terms of 16S rRNA gene sequence, other molecular taxonomy techniques have to be used to differentiate and identify them (Coenye and Vandamme, 2003; Spilker et al., 2009; Peeters et al., 2013). Hence, DDH and MLSA were performed in this study. The DDH results which showed that the DNA–DNA relatedness value between strain MSh1^T^ and its closely related neighbors (based on 16S rRNA phylogenetic analysis) were below the 70% threshold value for species delineation (Wayne et al., 1987). This was further supported by the average concatenated allele sequence divergence (obtained from the MLSA analysis) of strain MSh1^T^ toward other Bcc species which were above the 3% threshold (Librado and Rozas, 2009). Moreover, based on the biochemical profile of strain MSh1^T^, it can be distinguished from most Bcc by its inability to assimilate N-acetyl-glucosamine. Furthermore, this study has used a combination of both polyphasic and genome comparison (via next generation sequencing) to determine the species delineation of strain MSh1^T^. For every technique used, each result revealed that strain MSh1^T^ is indeed a novel species within the Bcc.

The genus *Burkholderia* is well known for producing a wide range of secondary metabolites which include siderophores (Asghar et al., 2011), antifungal (Lu et al., 2009; Tawfik et al., 2010), antibacterial (El-Banna and Winkelmann, 1998; Mitchell and Teh, 2005; Mitchell et al., 2008), antitumor (Klausmeyer et al., 2011; He et al., 2014), and exotoxins (Jeong et al., 2003; Partida-Martinez and Hertweck, 2007). Secondary metabolites are compounds which are non-essential for microbial growth or reproduction but provide diverse survival functions in nature (Martin et al., 2005). Many of these secondary metabolites are regulated by complex synthesis mechanisms within the bacteria itself which include the PKS and NRPS. The assembly of a polyketide via the PKS system begins by priming the starter molecule to a catalytic domain, followed by chain elongation with extender units by different kind of enzymes. As for the NRPS system, a NRP will firstly be generated and then linked with other molecules for instance a phosphate group, methyl group or fatty acids (Donadio et al., 2007; Amoutzias et al., 2008). In this present study, antiSMASH was used to annotate the secondary metabolite biosynthetic gene clusters present in the draft genome of strain MSh1^T^. It was found that strain MSh1^T^ has a higher number of secondary metabolite biosynthetic gene clusters as compared to other known antimicrobial producing Bcc species, for example *B. cepacia* and *B. pyroccinia* (Souza and Raaijmakers, 2003). Both PKS and NRPS gene clusters are present in the genome, indicating the antimicrobial compounds produced might be assembled by either of these two gene clusters. Moreover, strain MSh1^T^ might be able to produce a more complex antimicrobial compound as a hybrid PKS-NRPS was annotated by antiSMASH. One example of such an antimicrobial compound is a lipopeptide class compound (Sorensen et al., 2014). Lipopeptides are antimicrobial compounds, known to disrupt the surface membrane charges of its target. Thus far, there have been only two reported lipopeptides (burkholdine and occidiofungin) produced by Bcc species and they only possess antifungal activity (Lu et al., 2009; Tawfik et al., 2010). Bacteriocins are small peptides with narrow-spectrum antimicrobial properties (Cotter et al., 2013). Capistruin, is an example of antimicrobial peptide produced by a *Burkholderia* species. Nevertheless, the antimicrobial activity is only limited to several Gram negative bacteria which include *Burkholderia* species, *Psuedomonas aeruginosa*, and *E. coli* (Knappe et al., 2008). The data obtained from antiSMASH provided a preliminary insight into the secondary metabolite gene clusters present in the genome of strain MSh1^T^ and it is certain that strain MSh1^T^ can indeed produce antimicrobial compounds. As many secondary metabolite biosynthetic gene clusters were present in the genome, the antimicrobial activity observed might be due to the presence of multiple compounds. Therefore, extraction of the antimicrobial compounds produced by strain MSh1^T^ was performed in this study.

Since the antimicrobial activity of strain MSh1^T^ was determined with agar overlay assay in the previous study (Ong et al., 2015), this showed that the antimicrobial compounds produced could be extracted from the agar itself. Therefore, agar extraction (solid-liquid extraction) was carried out. This technique was adopted from a study conducted by Isnansetyo and Kamei (2003) in which they successfully extracted an antimicrobial compound produced by *Pseudoalteromonas phenolica* cultured on marine solid media. It was shown that *E. faecalis* ATCC 29212 and *E. faecalis* ATCC 700802 were highly sensitive to the antimicrobial compounds with MIC-value of 0.020 mg/ml and 0.039 mg/ml, respectively, as compared to *S. aureus* ATCC 29213 (0.313 mg/ml), *S. aureus* ATCC 700699 (0.156 mg/ml) and *E. coli* ATCC 25922 (2.500 mg/ml) (consistent with Ong et al., 2015). To the best of our knowledge, there has only been one antimicrobial compound with inhibitory effect against Gram positive bacteria and fungus (pyrrolnitrin), isolated from *Burkholderia* species, and based on the PCR performed in this study, the *prnD* gene was not found in strain MSh1^T^ which further substantiate that it might be producing other types of antimicrobial compounds. Furthermore, the genes that code for *prnA, prnB, prnC* and *prnD* were absent from the draft genome of strain MSh1^T^.

Sequential solvent fractionation was performed to fractionate and partially purify, at the same time removing impurities present in the crude methanol extract. A similar method was adopted from Tawfik et al. (2010) to isolate antifungal burkholdines produced by *B. ambifaria* 2.2N. In this study, four different solvents were used: hexane (HEX), dichloromethane (DCM), ethyl acetate (EtOAc) and water. HEX is a non-polar solvent commonly used to remove non-polar compounds from the crude extract. DCM and EtOAc both have middle-range polarity, thus is able to attract a wider range of compounds with different polarity (Moreau et al., 2003). Water being the most polar solvent will retain any compounds with highly polarity (Wang and Weller, 2006). It was shown that the HEX fraction had no antimicrobial activity against all test microorganisms up to 5 mg/mL. The DCM fraction had the best antimicrobial activity against all the test microorganism strains (when compared with the EtOAc and water fractions) as the MIC-values were comparable or similar to the crude methanol extract (Table 3). Thus, further purification was conducted on the DCM fraction using C_18_ which led to the isolation of Compound 1. Chromatographic analysis of Compound 1 on a TLC plate with chloroform-acetic acid-ethanol at 90:5:2.5 (v/v) as the mobile phase showed one spot (R_f_ 0.35) with yellow-green fluorescent band that turned red-brown after spraying with the iron reagent. Analysis of Compound 1 by analytical HPLC indicated that the compound contained one peak with three maxima at 210, 270, and 320 nm. The mass of Compound 1 (*m/z* 325, [*M*+H]^+^) was determined by liquid chromatography-electrospray ionization-mass spectrometer (LC-ESI-MS). The ESI-MS indicated that the molecular formula of the compound was C_14_H_16_N_2_O_3_S_2_. The interpretation of ESI-MS and UV spectrum of Compound 1 were found to be identical to pyochelin which was in good agreement with previous literature (Cox et al., 1981; Adler et al., 2012) (Figures S1, S2).

**Figure 5:**
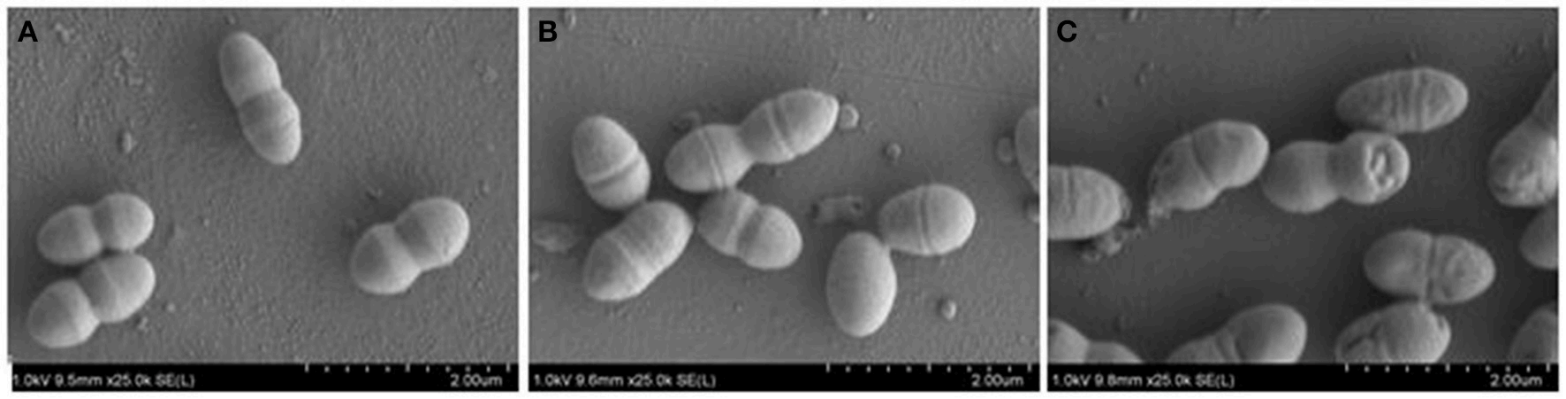
**FE-SEM images of *E. faecalis* ATCC 700802 showing (A)** negative control, **(B)** treated with MIC of Compound 1 and **(C)** treated with 100 μg/ml of chloramphenicol (positive control). Images were taken under 5000x magnification at 1 kV.

Pyochelin is a type of siderophore commonly produced by the genus *Pseudomonas* (Cox et al., 1981; Buysens et al., 1996; Lim et al., 2016). Siderophores can solubilize ferric ion, hence is an iron chelating growth factor for many bacteria. Thus far, only three *Burkholderia* species (*B. arboris, B. cenocepacia* and *B. contaminans*) have been known to produce pyochelin (Dang et al., 2011; Schwagner et al., 2012; Deng et al., 2015). As pyochelin is a siderophore, its antimicrobial properties were not extensively studied. Pyochelin can inhibit or kill bacteria by catalyzing the generation of reactive oxygen species (ROS) (Adler et al., 2012). However, this is bacteria dependent as shown in Table 4. The Gram negative bacteria (typically the *Enterobacteriaceae*) were found to be resistant to Compound 1 (pyochelin) and this is consistent with the study conducted by Adler et al. (2012). The resistant profile shown by the *Enterobacteriaceae* was due to the production of catecholate siderophores such as enterobactin. These siderophores can act as hydrogen atom donors and efficiently terminate radical chain reactions; hence rendering pyochelin ineffective. The inhibitory effect of pyochelin on the four *S. aureus* and three *E. faecalis* strains might due to presence of ROS generated by pyochelin. Nevertheless, no literature has reported on the downstream effect of the ROS produced by pyochelin, as it might target the DNA/RNA replication, electron transport chain or bacterial cell membrane. Moreover, the nutrient availability of these strains might be compromised as pyochelin can chelate the vital metal ions present in the environment. Consequently, these sensitive strains might be starved and ultimately led to death, as they lack the receptors to mediate the entry of metal-bounded pyochelin into the bacterial cells. Hence as a preliminary study, we investigated the effect of pyochelin on the cellular morphology of *E. faecalis* ATCC 700802 via SEM. Pyochelin did not affect the cellular morphology of *E. faecalis* ATCC 700802, when compared to the positive control in which the morphology of the bacteria were distorted when treated with 100 μg/ml of chloramphenicol (Figure 5). This result supports that the ROS generated by pyochelin might target the intracellular components of *E. faecalis* ATCC 700802. Further work on the effect of pyochelin on the DNA/RNA replication will be validated via qPCR in the future. Moreover, the antimicrobial activity of pyochelin was not only restricted to normal strains of *S. aureus* and *E. faecalis*, but it also affects the antimicrobial-resistant strains, for instance methicillin-resistant *S. aureus* ATCC 700699, methicillin-resistant *S. aureus* ATCC 43300 and vancomycin-resistant *E. faecalis* ATCC 700802. However, this has to be validated by increasing the number of test strains used. Additional work is on-going to further elucidate the downstream effect of pyochelin on these sensitive strains, and also to determine its potential synergistic action with antibiotics. Nevertheless, this study has provided an insight that the infamous pyochelin might potentially be used to treat infections caused by ARB.

**Table 4:** **The minimum inhibitory concentration (MIC) of Compound 1 (pyochelin) against 18 test microorganisms**.

**Gram stain**	**Bacteria strains**	**MIC (μg/ml)**
Gram positive	*Bacillus cereus* ATCC 14579	>100.00
	*Bacillus subtilis* ATCC 8188	>100.00
	*Enterococcus faecalis* ATCC 700802	3.13
	*Enterococcus faecalis* ATCC 29212	3.13
	*Enterococcus faecalis* JH-22	3.13
	*Staphylococcus aureus* ATCC 700699	6.26
	*Staphylococcus aureus* ATCC 43300	6.26
	*Staphylococcus aureus* ATCC 6538P	6.26
	*Staphylococcus aureus* ATCC 29213	6.26
Gram negative	*Aeromonas hydrophila* ATCC 49140	>100.00
	*Escherichia coli* ATCC 25922	>100.00
	*Klebsiella pneumoniae* ATCC 10031	>100.00
	*Proteus mirabilis* ATCC 49140	>100.00
	*Proteus vulgaris* IMR	>100.00
	*Pseudomonas aeruginosa* ATCC 10145	>100.00
	*Pseudomonas aeruginosa* ATCC BAA-47	>100.00
	*Salmonella* Typhimurium ATCC 14028	>100.00
	*Shigella flexneri* ATCC 12022	>100.00

Based on the results of phylogenetic analysis, DDH, MLSA, GGDC, ANI, phenotypic, chemotaxonomic and biochemical characterization, strain MSh1^T^ was assigned to a novel species in the genus *Burkholderia*, for which the name *Burkholderia paludis* sp. nov. is proposed.

### Description of *Burkholderia paludis* sp. nov.

*Burkholderia paludis* (pa.lu.dis. L. gen. n. *paludis* of the swamp). Cells are Gram negative, facultative anaerobic, motile rods, about 0.6–0.8 μm wide and 1.6–2.1 μm long. Colonies are regular, circular, convex, translucent, moist and 1.0–3.0 mm in diameter after 2 days of cultivation at 30°C on NA. Grows at 15–40°C (optimum 30°C) and pH 4.0–10.0 (optimum pH 7.0) in NB. Grows at 0–2.5% NaCl (optimum without NaCl). Cells are positive for oxidase, glucose fermentation, arginine dihydrolase, urease, esculin hydrolysis and assimilation of glucose, but negative for nitrate/nitrite reduction, indole production, gelatin hydrolysis, β-galactosidase, assimilation of arabinose, mannose, mannitol, N-acetyl-glucosamine, maltose, potassium gluconate, capric acid, adipic acid, malate, trisodium citrate and phenylacetic acid (API 20NE).

In API ZYM-tests, positive for alkaline phosphatase, esterase (C4), esterase lipase (C8), leucine arylamidase, acid phosphatase, naphthol-AS-BI-phosphohydrolase, β-glucosidase and n-acetyl-β-glucosaminidase, but negative for valine arylamidase, cystine arylamidase, trypsin, a-chymotrypsin, α-galactosidase, β-galactosidase, β-glucoronidase, α-glucosidase, αmannosidase and α-fucosidase. The following carbon sources are utilized in the API 50CH: glycerol, erythritol, D-arabinose, L-arabinose, ribose, D-xylose, galactose, D-glucose, D-fructose, D-mannose, dulcitol, inositol, mannitol, sorbitol, α-methyl-D-glucoside, arbutin, esculin, salicin, cellobiose, maltose, lactose, saccharose, trehalose, D-turanose, D-xylose, D-tagatose, D-fucose, L-fucose, D-arabitol and L-arabitol; the other substrates, L-xylose, adonitol, α-methyl-D-xiloside, L-sorbose, rhamnose, α-methyl-D-mannoside, α-methyl-D-glucosamine, amygdalin, melibiose, inulin, melezitose, D-raffinose, amidon, glycogen, xylitol, β-gentiobiose, gluconate, 2-keto-gluconate and 5-keto-gluconate, are not utilized.

The fatty acid profile is composed mainly of C_16:0_ (31.7%), C_17:0_ cyclo (26.6%), and C_19:0_ cyclo ω8c (16.1%). The major compounds in the polar lipid profile are phosphatidylglycerol, phosphatidylethanolamine and diphosphatidylglycerol. The predominant ubiquinone is Q-8. Strain MSh1^T^ is able to produce antimicrobial compounds active against four strains of *S. aureus* and three strains of *E. faecalis*.

The type strain is MSh1^T^ (= DSM 100703^T^ = MCCC 1K01245^T^), isolated from surface peat from the Southeast Pahang tropical peat swamp forest reserve, Malaysia. The 16S rRNA and MLSA gene sequence of strain MSh1^T^ has been deposited in GenBank/EMBL/DDBJ under the accession number KT159931 and KU301866–301872, respectively.

## Accession numbers

The GenBank/EMBL/DDBJ accession number for the 16S rRNA gene sequence of strain MSh1^T^ is KT159931. The GenBank/EMBL/DDBJ accession number for the MLSA gene sequences of strain MSh1^T^ are KU301866–301872.

## Author contributions

KSO performed the laboratory experiments, data analysis and the manuscript write-up. SML supervised the entire study. LHL and YLC co-supervised the study. SML, KSO and YKA contributed to the experimental designs. LHL and KSO contributed to the polyphasic taxonomy. CMY, SML and YKA apprehended the idea of bioprospecting in tropical peat swamp forest. All authors proofread and reviewed the manuscript.

### Conflict of interest statement

The authors declare that the research was conducted in the absence of any commercial or financial relationships that could be construed as a potential conflict of interest.

## Abbreviations

Bcc: Burkholderia cepacia complex
MLSA: multilocus sequence analysis.
