# Supplementary material for: Burkholderia paludis sp. nov., an Antibiotic-Siderophore Producing Novel Burkholderia cepacia Complex Species, Isolated from Malaysian Tropical Peat Swamp Soil
Source: Front Microbiol. 2016 Dec 21;7:2046. doi: 10.3389/fmicb.2016.02046 (PMC5174137; doi:10.3389/fmicb.2016.02046)
Supplement: Supplementary file 1 [file DataSheet1.DOCX]

**TABLE S1** Allelic profile of seven house-keeping genes of strain MSh1^T^ compared with 30 Bcc type strains. Value represent position of the gene in their respectively allele.

| *Burkholderia* type strains | Allellic profile | | | | | | |
| --- | --- | --- | --- | --- | --- | --- | --- |
|  | *atp*D | *glt*B | *gyr*B | *rec*A | *lep*A | *pha*C | *trp*B |
| *Burkholderia* *pyrrocinia* LMG 14191^T^ | 70 | 88 | 250 | 108 | 98 | 28 | 86 |
| *Burkholderia* *stabilis* LMG 14294^T^ | 26 | 18 | 14 | 21 | 70 | 10 | 16 |
| ***Burkholderia* *paludis* MSh1^T^** | **302** | **11** | **50** | **350** | **288** | **249** | **387** |
| *Burkholderia* *cenocepacia* IIIC LMG 19230^T^ | 65 | 49 | 41 | 47 | 33 | 36 | 44 |
| *Burkholderia* *cepacia* ATCC 25416^T^ | 5 | 4 | 44 | 4 | 4 | 4 | 48 |
| *Burkholderia* *metallica* AU0553^T^ | 239 | 189 | 268 | 187 | 202 | 153 | 242 |
| *Burkholderia* *contaminans* LMG 23361^T^ | 64 | 80 | 76 | 89 | 105 | 97 | 70 |
| *Burkholderia* *lata* 383^T^ | 63 | 46 | 38 | 44 | 30 | 33 | 42 |
| *Burkholderia* *arboris* LMG 14939^T^ | 60 | 43 | 36 | 42 | 27 | 72 | 72 |
| *Burkholderia* *seminalis* R-24196^T^ | 203 | 161 | 386 | 144 | 286 | 123 | 240 |
| *Burkholderia* *cenocepacia* IIID CCUG 46446^T^ | 148 | 172 | 216 | 161 | 24 | 30 | 38 |
| *Burkholderia* *cenocepacia* IIIA J2315^T^ | 15 | 11 | 9 | 14 | 11 | 6 | 12 |
| *Burkholderia* *cenocepacia* IIIB R-52732^T^ | 16 | 98 | 603 | 365 | 417 | 41 | 404 |
| *Burkholderia* *ambifaria* AMMD^T^ | 35 | 25 | 123 | 98 | 103 | 59 | 49 |
| *Burkholderia* *anthina* R-4183^T^ | 43 | 33 | 27 | 33 | 20 | 21 | 30 |
| *Burkholderia* *diffusa* R-15930^T^ | 98 | 42 | 68 | 87 | 53 | 50 | 41 |
| *Burkholderia* *latens* R-5630^T^ | 96 | 169 | 209 | 150 | 176 | 99 | 150 |
| *Burkholderia* *vietnamiensis* LMG 10929^T^ | 27 | 19 | 15 | 23 | 35 | 56 | 17 |
| *Burkholderia* *pseodmultivorans* CCUG 62895^T^ | 150 | 204 | 205 | 250 | 171 | 163 | 304 |
| *Burkholderia* *dolosa* LMG 18943^T^ | 30 | 21 | 18 | 24 | 72 | 13 | 20 |
| *Burkholderia* *multivorans* ATCC BAA-247^T^ | 11 | 60 | 117 | 81 | 37 | 86 | 97 |
| *Burkholderia* *ubonensis* CIP 107078^T^ | 137 | 229 | 200 | 216 | 243 | 177 | 153 |
| *Burkholderia* *oklahomensis* HI4355^T^ | 270 | 296 | 434 | 279 | 332 | 236 | 313 |
| *Burkholderia* *thailandensis* E264^T^ | 253 | 278 | 411 | 261 | 311 | 220 | 277 |
| *Burkholderia* *mallei* ATCC 23344^T^ | 95 | 117 | 128 | 46 | 32 | 34 | 43 |
| *Burkholderia* *pseudomallei* K96243^T^ | 95 | 116 | 128 | 45 | 31 | 34 | 43 |
| *Burkholderia* *gladioli* HI2137^T^ | 223 | 309 | 417 | 265 | 318 | 225 | 312 |
| *Burkholderia* *glumae* AU12450^T^ | 252 | 305 | 394 | 251 | 309 | 219 | 269 |
| *Burkholderia* *tropica* AU15822^T^ | 255 | 280 | 413 | 263 | 313 | 223 | 279 |
| *Burkholderia* *fungorum* AU 12699^T^ | 249 | 275 | 406 | 256 | 305 | 216 | 274 |
| *Burkholderia* *glathei* HI4344^T^ | 264 | 291 | 429 | 274 | 326 | 233 | 288 |
