# Supplementary material for: Burkholderia paludis sp. nov., an Antibiotic-Siderophore Producing Novel Burkholderia cepacia Complex Species, Isolated from Malaysian Tropical Peat Swamp Soil
Source: Front Microbiol. 2016 Dec 21;7:2046. doi: 10.3389/fmicb.2016.02046 (PMC5174137; doi:10.3389/fmicb.2016.02046)
Supplement: Supplementary file 2 [file DataSheet2.DOCX]

**TABLE S2** Percentage of divergence of concatenated allele sequences between strain MSh1^T^ with 30 Bcc type strains.

| *Burkholderia* type strains | Divergence (%) |
| --- | --- |
| *Burkholderia pyrrocinia* LMG 14191^T^ | 5.81 |
| *Burkholderia stabilis* LMG 14294^T^ | 5.23 |
| ***Burkholderia paludis* MSh1^T^** | **-** |
| *Burkholderia cenocepacia* IIIC LMG 19230^T^ | 6.87 |
| *Burkholderia cepacia* ATCC 25416^T^ | 6.44 |
| *Burkholderia metallica* AU0553^T^ | 6.71 |
| *Burkholderia contaminans* LMG 23361^T^ | 6.08 |
| *Burkholderia lata* 383^T^ | 5.50 |
| *Burkholderia arboris* LMG 14939^T^ | 6.51 |
| *Burkholderia seminalis* R-24196^T^ | 6.70 |
| *Burkholderia cenocepacia* IIID CCUG 46446^T^ | 6.63 |
| *Burkholderia cenocepacia* IIIA J2315^T^ | 6.59 |
| *Burkholderia cenocepacia* IIIB R-52732^T^ | 7.11 |
| *Burkholderia ambifaria* AMMD^T^ | 6.99 |
| *Burkholderia anthina* R-4183^T^ | 8.27 |
| *Burkholderia diffusa* R-15930^T^ | 7.11 |
| *Burkholderia latens* R-5630^T^ | 7.74 |
| *Burkholderia vietnamiensis* LMG 10929^T^ | 8.99 |
| *Burkholderia pseodmultivorans* CCUG 62895^T^ | 7.46 |
| *Burkholderia dolosa* LMG 18943^T^ | 8.51 |
| *Burkholderia multivorans* ATCC BAA-247^T^ | 8.14 |
| *Burkholderia ubonensis* CIP 107078^T^ | 8.18 |
| *Burkholderia oklahomensis* HI4355^T^ | 11.31 |
| *Burkholderia thailandensis* E264^T^ | 11.35 |
| *Burkholderia mallei* ATCC 23344^T^ | 10.93 |
| *Burkholderia pseudomallei* K96243^T^ | 10.89 |
| *Burkholderia gladioli* HI2137^T^ | 15.66 |
| *Burkholderia glumae* AU12450^T^ | 10.01 |
| *Burkholderia tropica* AU15822^T^ | 15.35 |
| *Burkholderia fungorum* AU 12699^T^ | 18.20 |
| *Burkholderia glathei* HI4344^T^ | 10.42 |
