# Supplementary material for: Burkholderia paludis sp. nov., an Antibiotic-Siderophore Producing Novel Burkholderia cepacia Complex Species, Isolated from Malaysian Tropical Peat Swamp Soil
Source: Front Microbiol. 2016 Dec 21;7:2046. doi: 10.3389/fmicb.2016.02046 (PMC5174137; doi:10.3389/fmicb.2016.02046)
Supplement: Supplementary file 3 [file DataSheet3.DOCX]

**TABLE S3** GGDC similarity values and ANI between strain MSh1^T^ to related Bcc species

| Bcc species | Wetlab DDH (% ± standard deviation) | GGDC (% Similarity ± confidence interval) | ANI (% ± standard deviation) |
| --- | --- | --- | --- |
| *Burkholderia arboris* R-24201^T^ | 29.0 ± 3.7 | n/a | n/a |
| *Burkholderia cenocepacia* J2315^T^ | 32.8 ± 2.2 | 42.40 ± 2.53 | 92.28 ± 3.33 |
| *Burkholderia lata* sp. 383^T^ | 19.5 ± 1.8 | 43.70 ± 2.54 | 92.55 ± 3.33 |

n/a: not available as the genome of *Burkholderia arboris* R-24201^T^ has not been sequenced
