# Supplementary figures and images for: Burkholderia paludis sp. nov., an Antibiotic-Siderophore Producing Novel Burkholderia cepacia Complex Species, Isolated from Malaysian Tropical Peat Swamp Soil
Source: Front Microbiol. 2016 Dec 21;7:2046. doi: 10.3389/fmicb.2016.02046 (PMC5174137; doi:10.3389/fmicb.2016.02046)

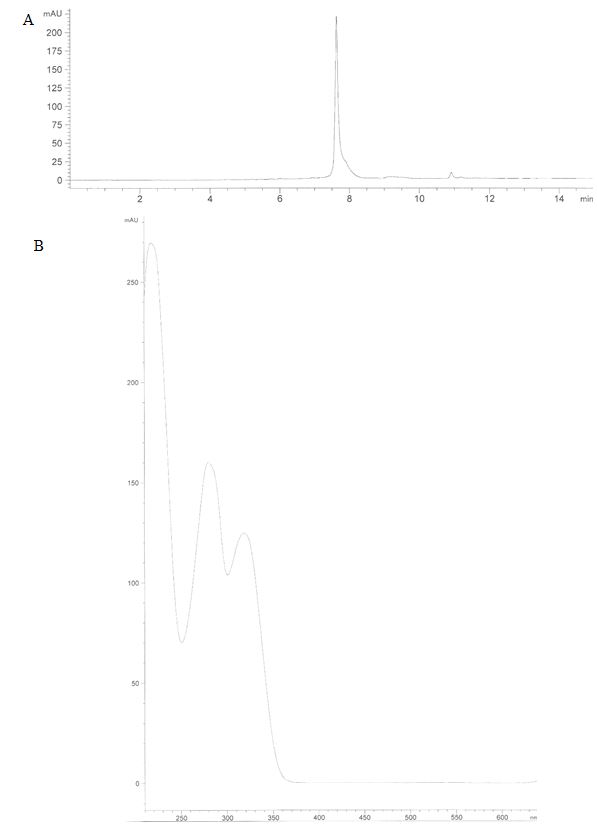


**FIG. S1**. (A) HPLC chromatogram and (B) UV spectra of Compound 1 (pyochelin)

Supplement: Supplementary file 4 [file DataSheet4.DOCX]
