# Supplementary material for: Burkholderia paludis sp. nov., an Antibiotic-Siderophore Producing Novel Burkholderia cepacia Complex Species, Isolated from Malaysian Tropical Peat Swamp Soil
Source: Front Microbiol. 2016 Dec 21;7:2046. doi: 10.3389/fmicb.2016.02046 (PMC5174137; doi:10.3389/fmicb.2016.02046)
Supplement: Supplementary file 5 [file DataSheet5.DOCX]

**FIG. S2**. LC-MS spectrum (M + H)^+^ and structure of Compound 1 (pyochelin)
